# Association between Maternal Exposure to Ambient Air Pollution and the Risk of Preterm Birth: A Birth Cohort Study in Chongqing, China, 2015–2020

**DOI:** 10.3390/ijerph19042211

**Published:** 2022-02-15

**Authors:** Wenzheng Zhou, Xin Ming, Yunping Yang, Yaqiong Hu, Ziyi He, Hongyan Chen, Yannan Li, Xiaojun Zhou, Ping Yin

**Affiliations:** 1 Department of Epidemiology and Biostatistics, School of Public Health, Tongji Medical College, Huazhong University of Science and Technology, Wuhan 430030, China; zhouwenzheng@126.com; 2 Chongqing Health Center for Women and Children, Chongqing 401147, China; 13638320537@163.com (X.M.); yunpingcqfy@163.com (Y.Y.); yaqiongcqfy@163.com (Y.H.); ziyicqfy@163.com (Z.H.); chenhongyan1119@163.com (H.C.); lyn93111906@163.com (Y.L.)

**Keywords:** preterm birth, air pollution, risk assessment, environmental exposure

## Abstract

Recent study results on the association between maternal exposure to ambient air pollution with preterm birth have been inconsistent. The sensitive window of exposure and influence level of air pollutants varied greatly. We aimed to explore the association between maternal exposure to ambient air pollutants and the risk of preterm birth, and to estimate the sensitive exposure time window. A total of 572,116 mother–newborn pairs, daily concentrations of air pollutants from nearest monitoring stations were used to estimate exposures for each participant during 2015–2020 in Chongqing, China. We applied a generalized additive model and estimated RRs and 95% CIs for preterm birth in each trimester and the entire pregnancy period. In the single-pollutant model, we observed that each 10 μg/m^3^ increase in PM_2.5_ had a statistically significant effect on the third trimester and entire pregnancy, with RR = 1.036 (95% CI: 1.021, 1.051) and RR = 1.101 (95% CI: 1.075, 1.128), respectively. Similarly, for each 10 μg/m^3^ increase in PM_10_, there were 2.7% (RR = 1.027, 95% CI: 1.016, 1.038) increase for PTB on the third trimester, and 3.8% (RR = 1.038, 95% CI: 1.020, 1.057) increase during the whole pregnancy. We found that for each 10 mg/m^3^ CO increases, the relative risk of PTB increased on the first trimester (RR = 1.081, 95% CI: 1.007, 1.162), second trimester (RR = 1.116, 95% CI: 1.035, 1.204), third trimester (RR = 1.167, 95% CI: 1.090, 1.250) and whole pregnancy (RR = 1.098, 95% CI: 1.011, 1.192). No statistically significant RR was found for SO_2_ and NO_2_ on each trimester of pregnancy. Our study indicates that maternal exposure to high levels of PM_2.5_ and PM_10_ during pregnancy may increase the risk for preterm birth, especially for women at the late stage of pregnancy. Statistically increased risks of preterm birth were associated with CO exposure during each trimester and entire pregnancy. Reducing exposure to ambient air pollutants for pregnant women is clearly necessary to improve the health of infants.

## 1. Introduction

In recent years, environmental pollution has become a global problem affecting human health. Since the start of 21st century, the literature in the field of epidemiology and clinical medicine have reported the hazardous effects of ambient air pollution on human health. Multiple studies indicate that air pollution may be related to adverse birth outcomes, including preterm birth, low birth weight, stillbirth and spontaneous abortion, etc. [1,2,3]. Both preterm birth and low birth weight are major adverse neonatal outcomes that are strongly related to infant mortality, and even severe ongoing adverse health effects reaching into childhood. A host of investigations have explored the association between maternal exposures to ambient air pollutants and preterm birth [4,5,6]. Still, thus far, the results of studies on the effects of maternal exposure during pregnancy have been inconsistent. The sensitive window of exposure and the influence level of air pollutants have varied greatly [6,7,8]. Further, some of the studies are ecological in nature or limited by a small sample size [9]. More research is needed to explore the ongoing risk of air pollutants on preterm birth.

The latest national cohort study conducted by Wang et al., in 30 provinces in China, reported that the estimated rate of preterm birth is around 8.0% at present [10]. That is a high rate worldwide, and further research on the impact factor and prevention is essential and needed in China. The studies on ambient air pollutants and preterm birth in China have delivered different conclusions in different regions [11,12,13]. Among the significant concerns that have been noted is the ambient air pollution associated with the increasing risk of preterm births. Yet, few studies have used birth cohorts and large sample sizes, because of the constraints of objective conditions. Liang and Guo et al. indicated that maternal PM_2.5_ exposure is a risk factor for both low birth weight and preterm birth in the Pearl River Delta region, based on a birth cohort study in nine Chinese cities [14,15]. Yuan et al. carried out a birth cohort study in Shanghai to explore the critical windows for maternal ambient air pollution and adverse birth outcomes [16]. However, there has been no similar research in the western region of China.

Chongqing, located in the west region of China, is the largest municipality in China, with a permanent population of 31 million. It is an industrial city located along the Yangtze River with 40 districts. Compared to other cities in China, Chongqing formerly had poor air quality and was known as the famous “Fog City”. Its air quality has been improving gradually since 2014 through strengthened environmental management by local government according to the annual environmental monitoring data. The monitoring work on ambient air pollutants in Chongqing has improved year by year, providing more research data than before. In total, about 1.5 million babies were born from January 2015 to December 2020 in Chongqing. The special air quality change trends in Chongqing offer a unique research environment that is different from European and American countries, and also give us a particular sample to use to study the effects of air pollution exposure on birth outcomes.

The purpose of this study was to explore the association between maternal exposure to ambient air pollutants (PM_2.5_, PM_10_, SO_2_, O_3_, NO_2_, CO) and the risk of preterm birth in Chongqing, China, and estimate the sensitive exposure time window.

## 2. Materials and Methods

### 2.1. Study Population

We conducted the study in Chongqing, China, based on a large retrospective cohort of live births from 2015 to 2020. All birth data were extracted from the birth registry data base for Chongqing. This birth certificate system collected all information about infants who were admitted to all midwifery clinics and hospitals in Chongqing. The birth information came from the medical records in each hospital and was submitted to the birth certificate system via a network system. The database contains maternal age, maternal residence address, the date of birth, birth weight, gestational age in week, etc. The environmental monitoring of Chongqing was concentrated in the central nine districts where the permanent resident population is about 10.34 million, accounting for 32.27% of the city during the study period. For rigorous matching of ambient air pollutants and birth outcome data, we also used birth data from the main nine districts.

To facilitate the comparison with previous similar studies, the birth data we used was limited to single live births for women with 20–42 completed weeks of gestation. Cases were excluded if they had missing data for exposure or birth outcome variables. Of those instances, we excluded births of either of the following: extremely low and high birth weight, the values being <350 g or >5000 g, respectively; the mother lived ≥10 km from the nearest monitor station. A total of 598,017 births that met the inclusion criteria were initially enrolled, about 572,116 births were included in the analyses after excluding the mother who lived ≥10 km from the nearest monitor station.

Preterm birth (PTB) was defined as a live birth with <37 weeks of gestational age. The date of birth and gestational age were used to establish the start and end dates of gestational exposure and estimate the exposure time during the stages of pregnancy. Trimesters were defined as the first trimester, 1–12 weeks, the second trimester, 13–27 weeks, and the third trimester, 28 weeks until birth [17].

Approval to conduct this study was obtained from the institutional ethical committee of Chongqing Health Center for Women and Children. The data used in this study were anonymous and were analyzed at an aggregate level. No individual identifiable information was available, and no participants were contacted.

### 2.2. Exposure Assessment

**Figure 1:**
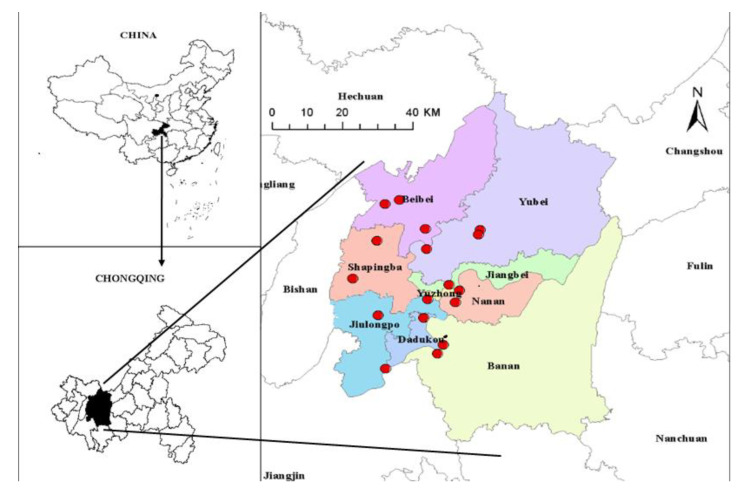
Locations for 17 monitoring sites (denoted by red circle) used to construct exposure estimates in Chongqing, China.

Air pollution data were all collected from the Chinese national urban air quality monitoring platform published by the China National Environmental Monitoring Station of the Ministry of Ecology and Environment (http://www.zhb.gov.cn, accessed on 25 March 2021). In this study, air pollution concentration data included PM_2.5_, PM_10_, SO_2_, CO, NO_2_ and O_3_. The concentrations of air pollutants PM_2.5_, PM_10_, O_3_, SO_2_ and NO_2_ are expressed in micrograms per cubic meter (μg/m^3^), and that for CO represented in milligrams per cubic meter (mg/m^3^), respectively. These air pollution data were measured at 17 air monitoring stations from 1 January 2015 to 30 December 2020 in 9 main districts of Chongqing, China (see Figure 1).

**Figure 2:**
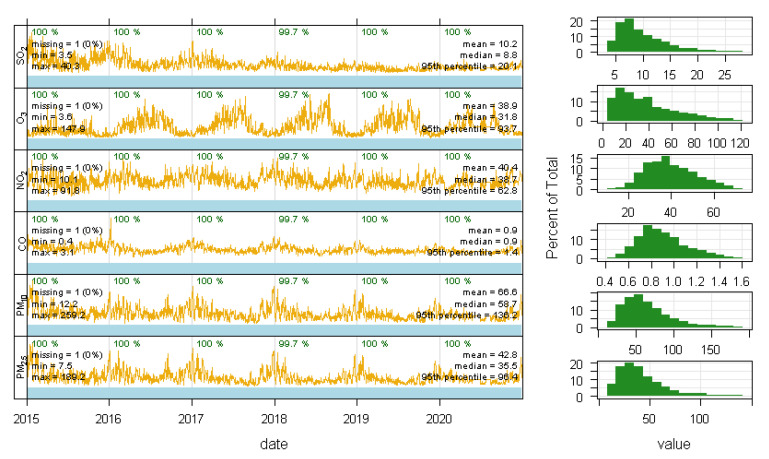
The plots in the left panel show the time series data. The daily mean values are shown in pale yellow scaled to cover the range in the data from zero to maximum daily value. The panels on the right show the distribution of each species using a histogram plot.

The daily concentration of ambient air pollutants gathered for this study was calculated according to the open data from the China National Environmental Monitoring Station of the Ministry of Ecology and Environment, and is shown in Figure 2.

Based on the detailed maternal residence address of each researched pregnant woman and the location of air monitoring stations, we obtained each latitude and longitude through the Amap open platform (https://lbs.amap.com/, accessed on 27 March 2021). Then, according to their exact spatial locations on the digital map, we calculated the distance between each maternal residence and the monitoring sites using ArcGIS (version 10.2). We used the data of the closest monitoring station to the maternal residence as the exposure value for each subject with a cut-off distance of 10 km, which is consistent with the related research literatures [4,18]. The start exposure time of pregnancy was estimated at the date of conception, which was calculated according to the time of the gestational week and the last menstruation of the woman [19]. We also calculated the mean concentrations during the entire pregnancy and every trimester based on daily monitoring data. This criterion was used to estimate overall and trimester-specific exposure values for all objects. It was beneficial that we could assign exposure values at the individual level, rather than distinct-level measurements [14].

To adjust for weather conditions, we obtained daily mean temperature (°C) and relative humidity (%) in Chongqing from the National Weather Data Sharing System (http://data.cma.cn/, accessed on 2 April 2021).

### 2.3. Statistical Analysis

To estimate the association between ambient air pollutant exposure and the risk of PTB in each exposure period, we applied a generalized additive model (GAM) [19,20,21]. The GAM model identifies nonlinear associations between variables. To control for the non-linear trend between preterm birth and time or weather conditions, we added time-dependent variables including calendar time, temperature and relative humidity via natural spline functions [22]. The GAM model was constructed for PTB in each trimester and the entire pregnancy period. The effects were examined by both single-pollutant and multiple-pollutant models. In this study, Model 1 meant a single-pollutant model, adjusted for covariates that included mean temperature and humidity; Model 2 was also a single-pollutant model, additionally adjusted the age of mother and father, weight of birth on the basis of Model 1; Model 3 was a multi-pollutant model, adjusted for covariates that included mean temperature and humidity, age of mother and father, weight of birth, and additionally adjusted for other air pollutant exposure.

The basic model can be described as follows:

(1) $$Log[E(Y_{t})]=\alpha +\beta Z_{t}+S(time,df)+S(temperature,\mathrm{df})+S(relativehumidity,\mathrm{df})+as.factor(Dow)$$

where *Log*[] is a link function; *t* is the observation day; α is the model intercept; β is the factor for each pollutant; $Z_{t}$ is the concentration of pollutants in day *t*; *S*() is the natural spline function; and Dow is dummy variable for day of week; *S*(*time*, df) is the conception time.

The sensitivity analyses were performed by altering the degrees of freedom for time (5–7 df/year), mean temperature (5–7 df), and relative humidity (2–4 df). We selected the df of the variables using the empirical values in the literature and minimizing the Akaike information criterion. Finally, the corresponding degree of freedom for time, temperature, and relative humidity in the spline function were 7, 3, and 3 in the model, respectively.

The associations were illustrated as relative risks (RRs) and 95% confidence intervals (95% CIs) for each 10 μg/m^3^ increase in the concentrations of PM_2.5,_ PM_10_, SO_2_, O_3_, NO_2_, and CO (mg/m^3^). We used R software (Version 4.1.0) for data analyses. The “splines” and “mgcv” packages were used to fit the GAM models. A probability value of <0.05 was considered as a statistical significance.

## 3. Results

### 3.1. Descriptive Statistics of Objects

Initially, a total of 598,018 births were collected from a retrospective birth cohort during the study period (1 January 2015–30 December 2020) in Chongqing. After a strict screening, an eligible dataset consisting of 572,116 mother–newborn pairs was finally analyzed for this study. The mothers in the study ranged in age from 18 to 37 years old, with an average age of 28.84. The mean (standard deviation) gestational age was 38.73 (1.49) weeks. Among them, 33,669 (5.88%) were PTB. Younger mothers and fathers had a relatively lower risk of PTB (see Table 1).

### 3.2. Descriptive Statistics of Air Pollutants

The characteristics of air pollution and their meteorological factors are summarized in Table 2. We found that there were seasonal trends for each ambient air pollutant, and that all have gradually decreased since 2017. The mean concentration of PM_2.5_ during the three trimesters and whole pregnancy were 42.86 μg/m^3^, 41.76 μg/m^3^, 41.43 μg/m^3^, and 41.62 μg/m^3^, respectively. Meanwhile, the mean concentrations of PM_10_ at the same time were 67.88 μg/m3, 66.49 μg/m^3^, 66.18 μg/m3, and 66.39 μg/m^3^, respectively. The mean concentration were 38.95 μg/m^3^ for NO_2_, 1.02 mg/m^3^ for CO, 9.73 μg/m^3^ for SO_2_, 38.45 μg/m^3^ for O_3_, 20.25 °C for apparent mean temperature and 75.25% relative humidity during the entire study period. Most pollutant correlations were positive except for O_3_. The correlations between air pollutants and meteorological factors were mostly negative, except for O_3_ and temperature, CO and humidity. A positive correlation between PM_2.5_ and PM_10_ (r = 0.910), and a negative correlation between PM_2.5_ and average daily temperature (r = −0.244) were observed (see Table 3).

### 3.3. Associations between Air Pollutants and PTB

The associations between PTB and maternal exposure to ambient air pollutants were calculated using the GAM method with single- and multiple-pollutant models. Adjusted RRs and 95% CIs for preterm birth for the entire pregnancy and every trimester are given in Table 4.

We observed statistically significant effects for each 10 μg/m^3^ increase in PM_2.5_ concentrations on the third trimester and the entire pregnancy. The RRs were 1.119 (95% CI: 1.101, 1.138) and 1.036 (95% CI: 1.021, 1.051) in Model 1 and Model 2 on the third trimester, respectively. On the entire pregnancy, the RRs were 1.185 (95% CI: 1.157, 1.215), 1.101 (95% CI: 1.075, 1.128) and 1.064 (95% CI: 1.031, 1.099) in Model 1, Model 2, and Model 3, respectively.

For each 10 μg/m^3^ increase in PM_10_, there were 10.7% (RR = 1.107; 95% CI: 1.094, 1.121) and 2.7% (RR = 1.027, 95% CI: 1.016, 1.038) increases for PTB on the third trimester in Model 1 and Model 2, respectively. There were 7.8% (RR = 1.078, 95% CI: 1.059, 1.098) and 3.8% (RR = 1.038, 95% CI: 1.020, 1.057) increases during the whole pregnancy in Model 1 and Model 2, respectively.

We found that for each 10 mg/m^3^ CO increase, the relative risk of PTB increased in every trimester and the whole pregnancy, with RR = 1.857 (95% CI: 1.733, 1.991), RR = 1.081 (95% CI: 1.007, 1.162) and RR = 1.094 (95% CI: 1.008, 1.188) in Model 1, Model 2, and Model 3, respectively, in the first trimester. In addition, it was associated with PTB in the second trimester in Model 1 with RR = 2.194 (95% CI: 2.035, 2.366), in Model 2 with RR = 1.116 (95% CI: 1.035, 1.204), and in Model 3 with RR = 1.117 (95% CI: 1.024, 1.218), respectively. Statistically significant increased RRs were consistently observed during the third trimester in Model 1 and Model 2 (RR = 1.758, 95% CI:1.635, 1.890, RR = 1.167, 95% CI:1.090, 1.250, respectively). Statistically significant association during the entire trimester of pregnancy was also visible in Model 1 and Model 2 (RR = 2.207, 95% CI: 2.031, 2.398, RR = 1.098, 95% CI:1.011, 1.192, respectively). As shown in Table 4, no statistically significant RR was found for PTB and maternal exposure to SO_2_ and NO_2_ on each trimester of pregnancy.

The results of the adjusted model for apparent mean temperature and humidity were most consistent with the single pollutant Model 1 (see Table 4). Altering the degrees of freedom for the adjustment of temperature and relative humidity did not substantially alter effect the estimates for the association between maternal exposure to air pollutants and PTB.

**Figure 3:**
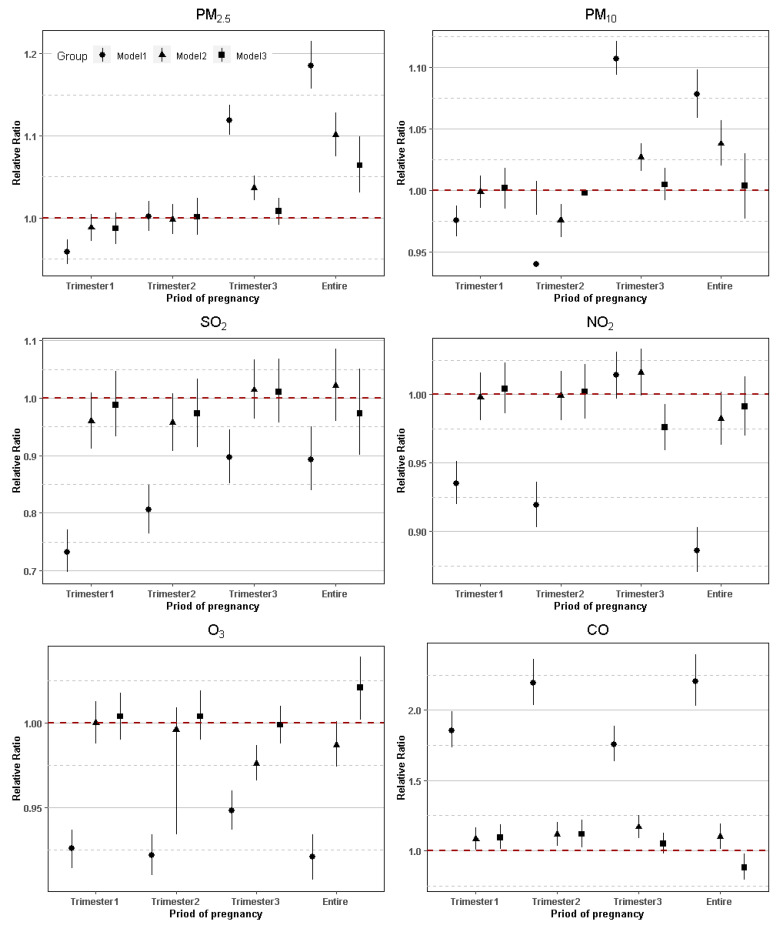
Adjusted RRs (95% CIs) for PTB associated with air pollutants during the different stages of pregnancy in Model 1–3. Note: Model 1: single-pollutant model, adjusted for covariates including mean temperature and humidity, which was represented by a circle; Model 2: single-pollutant model, adjusted for covariates including mean temperature and humidity, age of mother and father, and weight of birth, which was represented by a triangle; Model 3: multi-pollutant model, adjusted for covariates including mean temperature and humidity, age of mother and father, weight of birth, and additionally adjusted for other air pollutants, which was represented by a square.

Overall, a larger statistically significant association between maternal exposure to air pollutants and PTB was observed for different pregnancy stages and concentrated in PM_2.5_, PM_10_ and CO. Forest plots of the RR values and 95% CIs for PTB associated with maternal exposure to six pollutants during different stages of pregnancy are shown in Figure 3.

## 4. Discussion

### 4.1. Summary of Results

In this large sample study of the Chongqing birth population from 2015 to 2020, we found that several ambient air pollutants were associated with a higher risk of PTB, thereby confirming and extending the results of the few previous studies undertaken in China. Using a total of 598,018 births, based on 572,116 mother–child pairs from the birth cohort, this study revealed that maternal exposure to PM_2.5_ and PM_10_ in the third trimester and entire pregnancy was associated with increased PTB. In addition, a positive association with PTB was linked to CO exposure during every trimester and also the entire pregnancy.

### 4.2. Comparisons with Other Studies Worldwide

The past decade has witnessed mounting attention on researching the association between maternal exposure to air pollutants and adverse birth outcomes. Many studies have demonstrated positive associations between ambient air pollution exposure and PTB [23,24,25,26], especially for PM_2.5_ and PM_10_. Lei Yuan and Ying Tian (2019) reviewed 42 studies on the association between maternal exposure to PM_2.5_ and PTB; among 18 studies selected, prenatal PM_2.5_ exposure has strong effects on PTB [27]. A comprehensive review (2018) of ambient air pollution and pregnancy outcomes with 96 articles indicated that exposure to particulate matter and O_3_ over entire pregnancy was significantly associated with higher risk for PTB [28]. Haidong Kan et al. (2020) conducted a birth cohort study in China which indicated the effects of prenatal exposure to ambient PM_2.5_ on PTB, and identified the late pregnancy stage as a sensitive time window for adverse birth outcomes [16]. Their results are similar to this study. Further, Xiao et al. (2018) reported that a 10 μg/m^3^ increase in PM_2.5_ was associated with a 27% increase in PTB in gap-filled satellite-based whole-pregnancy of 132,783 singleton live births during 2011–2014 in Shanghai [17]. Although there were differences in ambient air pollutant concentration and exposure time window, as well as the specific OR or RR values, most of the literature reported statistically significant effects of maternal exposure to ambient air pollutants on PTB. However, a research of world health organization global survey on maternal and perinatal health [29] for 22 countries, from 2004 to 2008, found non-significant associations of PTB with PM_2.5_ for any pregnancy period. More than a time-series analysis and case–control study, the size of our study population and the mode of individual assessment based on the GAM models of birth cohort allowed us to assess the associations more accurately. It suggested that maternal exposure to the high concentrations level of gaseous air pollutants of PM_2.5_ and PM_10_ in the late pregnancy stage may stimulate premature births.

We found that for each 10 mg/m^3^ CO increases, the relative risk of PTB increased in every trimester and also the whole pregnancy. As with most literature reporting that maternal exposure to PM_2.5_ and PM_10_ is associated with PTB, there are relatively few reports about the association between CO and PTB. Juan Chen et al. (2021) carried out a birth cohort study of 10,960 pregnant women with singleton live births in Tianjin, China, reporting that maternal exposure to PM_2.5_, PM_10_, NO_2_, SO_2_ and CO during the before pregnancy period, specific trimesters and the entire pregnancy were associated with increased risks of PTB [7]. Zhengmin Qianet al. (2016) found 15% (OR = 1.15, 95% CI: 1.11, 1.19) increases in the risk of PTB with each 100μg/m^3^ increase in CO concentration during the pregnancy period [30]. On the contrary, a study conducted in Italy of air pollution impact on pregnancy outcomes reported exposure to CO and SO_2_ seemed to postpone delivery [31]. The results in the current study give reliable evidence that maternal exposure to CO increases risk of PTB with lager RR values (see Table 4).

No statistically significant RR was found for PTB and maternal exposure to SO_2_ and NO_2_ on each trimester of pregnancy. That may be related to the relative low concentration level of SO_2_ in Chongqing (see Table 2) compared to other industrial cities. The concentration of SO_2_ in Chongqing is around 10μg/m^3^ these years, while it is as high as 30–40μg/m^3^ in common industrial cities [12]. There was negligible evidence for the associations for O_3_. In fact, null or slightly inverse associations between the maternal exposure to air pollution and PTB were observed for both NO_2_ and O_3_ in the current studies [32,33,34]. Among the similar studies, negative or absent associations were not uncommon.

### 4.3. Analysis of Sensitive Exposure Windows

In our study, pregnancy exposure stage was divided into three equal parts according to gestational weeks similar to a lot of the other research [35,36,37]. Statistically significant increased RRs were consistently observed for PTB and maternal exposure to PM_2.5_ and PM_10_ during all stages of pregnancy. Consistent with a few of the previous studies [15,36], we observed that the third trimester and entire pregnancy might be more sensitive exposure windows for PM_2.5_ and PM_10_. Lavigne et al. (2018) analyzed 818,400 pairs of mothers and live births living in Ontario, Canada. They suggested there were impacts of prenatal exposure to ambient PM_2.5_ on PTB during the third trimester that with an OR of 1.01 (95% CI: 1.01, 1.02) [36]. Zhijiang Liang et al. conducted a study with 1,455,026 singleton births during 2014–2017 in nine cities of Guangdong, China, and found that PM_2.5_ was significantly associated with PTB in the second and the third trimesters of pregnancy, with relatively stronger effects of PM_2.5_ were observed on the third trimester (HR = 1.07, 95% CI: 1.06, 1.08) for each 10 μg/m^3^ increase in PM_2.5_ concentrations [15]. Another birth cohort conducted in Ohio, US, identified the third trimester as the most significant critical window for gestational PM_2.5_ exposure on PTB [38]. Similarly, a meta-analysis was conducted and reported [39] that the pooled OR for PM_2.5_ exposure (per 10 μg/m^3^ increment) during the entire pregnancy on PTB was 1.13 (95% CI: 1.03, 1.24) in 13 studies with a significant heterogeneity (Q = 80.51, *p* < 0.001), and was 1.08 (95% CI: 0.99, 1.17) in the third trimester.

However, other literature reported that the statistically significant exposure windows were the first or second trimester of pregnancy [28]. A birth cohort study in Wuhan, China, indicated the strongest effect for PTB was found in the second trimester for PM_2.5_, PM_10_, and CO [30]. On the other hand, the studies with different staging methods showed different results. For example, several researchers divided pregnancy into months or quarters to evaluate the association between maternal exposure and outcomes [32,40,41]. The exposure window difference of these research results maybe due to study design, air pollution level, regional disparity, composition of PM, and sample size, etc.

So far, only a few studies have evaluated the association of maternal exposure to ambient air pollution and risk of PTB in China. The evidence regarding sensitive exposure windows is still insufficient. Disagreement remained about whether the relative risk of exposure–response relationships varied in different exposure time windows. This study is based on a large sample birth cohort and individual-level exposure data and provided a more refined assessment and more credible results. Specially, in order to reduce the bias caused by the heterogeneity as much as possible, we applied GAM model to handle the hierarchy of data and, thus, obtained reliable trimester-specific exposure effects.

The biological mechanisms associated with PTB and air pollution has been researched a great deal in recent decades. The hypothesized mechanisms include direct toxic effects, inflammation and oxidative stress, placental vasoconstriction, DNA damage, and direct effects on the autonomic nervous system, etc. [42,43]. A recent epidemiologic study has also demonstrated that exposure to PM_2.5_ pollution is associated with changes in fetal thyroid hormone levels in the third trimester, which account partly for PTB and low birth weight [44]. A high risk of maternal exposure to PM_2.5_ and PM_10_ for PTB in the late pregnancy stage may be due to a cumulative or acute effect. The other possible mechanism of CO exposure may be similar to preeclampsia [45]. CO may induce hypoxia because it displaces oxygen from hemoglobin, forming carboxyhemoglobin. Even fairly low maternal carboxyhemoglobin concentrations can reduce oxygen transfer across the placenta. Lack of oxygen in the placenta can lead to premature birth. Our study does not focus on mechanisms, and greater understanding of exposure effect during the critical windows needs more targeted research.

### 4.4. Adjusted for Covariates in Single and Multi-Pollutant Model

To investigate the PTB risk associated with air pollutants in different pregnancy stages, we applied GAM using three models. Model 1 and Model 2 were single-pollutant models adjusted for covariates. Model 1 adjusted mean temperature and humidity; Model 2 adjusted mean temperature and humidity, age of mother and father, and weight of birth; Model 3 was a multi-pollutant model, adjusted for covariates as with Model 2, and additionally adjusted for other air pollutants exposure. Many studies have used the practice of control covariates [24,46,47]. Their results showed that after controlling for the impact of confounding factors, there were significant associations between PTB and air pollutants. In this current study, the statistically significant results found in Model 2 were similar to Model 1. However, positive results of the multi-pollutant model were quite rare compared to other models. The observed associations between PM_2.5_, PM_10_ and PTB also seemed to be confounded by other air pollutants according to the results. The models without adjustment for mean temperature and humidity were similar to Model 1 (Supplementary Table S1).

We also found high correlation coefficients between the average concentrations of air pollutants, for example, the correlation between PM_2.5_ and PM_10_ (correlation coefficient r = 0.910), PM_2.5_ and CO (r = 0.546), and PM_10_ and CO (r = 0.527). Thus, a multi-pollutant model could result in unstable estimates, due to high collinearity. The other possible reason might be that these air pollutants may have similar biological mechanisms on the effects of birth outcomes.

### 4.5. Assets and Limitations

Our study possessed several assets. We conducted a retrospective birth cohort study in Chongqing, China, and collected an eligible dataset consisting of 572,116 mother–newborn pairs from 2015–2020. It provided a rare, large sample-size study. Chongqing is a huge industrial city in the western region of China, with a permanent population of 31 million. The changing trend of air quality of Chongqing provides unique characteristics for studying the effects of air pollutants exposures to birth outcomes. There may be differences between our results and those in other countries or regions, and these may provide valuable reference for future study. Furthermore, we applied a generalized additive model (GAM) and assigned exposure values at the individual level for every day. The benefit was that we were able to assign exposure time that was more precise, and also make the best use of information, rather than using distinct-level measurements.

This study also has some limitations. First, like most of the previous studies, exposure measurement errors were inevitable because we estimated air pollution exposure values by using the information of air monitor stations nearest to maternal residence as proxies for personal exposure. If a mother lives next to the emission source but does not have any ground monitors around her, the mother will be assigned a low pollution level. Fortunately, our study has a large sample size. Second, we restricted our exposure analysis to mothers who lived within 10 km of a monitoring station. We were unable to assess whether these women moved during their pregnancies. The 10 km buffer radius around the stations was also an imprecise estimate of daily exposure. The human mobility issue is not considered during the exposure assessment, which creates uncertainty in the assessment results (resulting in over- and under-estimates) [48]. Third, we did not consider other potential risk factors due to data unavailability. For example, parental education, income levels or socioeconomic levels, maternal nutritional status, complications of pregnancy and behavioral information, etc., may all confound the observed associations in this study. However, previous studies found the effect estimates changed little with and without adjustment for the above risk factors [49]. Fourth, several of the pollutants were correlated, so related variables in a model might lead to modeling problems that hindered drawing meaningful conclusions from the results. Additionally, we did not get any data on components of particulate matter, the different components of which may elicit different categories of birth outcomes. Future research should focus on a hierarchical analysis of air pollution and biological mechanisms.

## 5. Conclusions

In summary, gestational exposure to ambient air pollutants significantly increases the risk for PTB. Our study indicates that maternal exposure to high levels of PM_2.5_ and PM_10_ during pregnancy may have increased preterm births, especially for women at the late stage of pregnancy. Maternal exposure to high levels of CO during each trimester and entire pregnancy may increase risk for PTB. Pregnant women should be encouraged to be aware of the potential hazards of ambient air pollution, pay more attention to local air quality and take appropriate protective measures. The public health department should formulate and implement environmental policies to reduce the risk of air pollution.

Further research including hierarchical analysis of personal monitoring data, stratified study of exposure time according to seasons, and methods for assessing exposure to mixtures of pollutants would strengthen the science in this field. Reducing exposure to ambient air pollutants for pregnant women is clearly necessary to improve the health of infants.

## Figures and Tables

**Table 1:** The descriptive summary of the general characteristics of live birth data.

Variables	PTB	Non-PTB	Total	*p* Value
	(*n* = 33,669, 5.88%)	(*n* = 538,447, 94.12%)	(*n* = 572,116)	
**Gestational age**	34.75 ± 1.81	38.97 ± 1.05	38.73 ± 1.49	<0.001
**Birth weight**	2459.07 ± 530.76	3328.69 ± 403.31	3277.52 ± 459.94	<0.001
**Maternal age**	30.00 ± 5.07	28.77 ± 4.93	28.84 ± 4.95	<0.001
<20 years	453 (1.35%)	7753 (1.44%)	8206 (1.43%)	<0.001
20–24 years	4218 (12.53%)	93,062 (17.28%)	97,280 (17%)	
25–29 years	11,249 (33.41%)	218,675 (40.61%)	229,924 (40.19%)	
30–34 years	11,531 (34.25%)	152,067 (28.24%)	163,598 (28.6%)	
≥35 years	6185 (18.29%)	66,426 (12.34%)	72,611 (12.69%)	
Missing	33 (0.9%)	464 (0.09%)	497 (0.09%)	
**Father age**	32.34 ± 5.39	31.06 ± 5.59	31.14 ± 5.62	<0.001
<20 years	96 (0.29%)	1644 (0.31%)	1740 (0.31%)	<0.001
20–24 years	2062 (6.12%)	46,922 (8.71%)	48,984 (8.74%)	
25–29 years	9068 (26.93%)	182,964 (33.98%)	192,032 (34.27%)	
30–34 years	11,258 (33.44%)	173,157 (32.16%)	184,415 (32.91%)	
≥35 years	10,241 (30.42%)	122,923 (22.83%)	133,164 (23.77%)	
Missing	944 (2.04%)	10,837 (2.0%)	11,781 (1.27%)	
**Season of conception**				
Spring	7993 (23.74%)	127,356 (23.65%)	135,349 (23.66%)	<0.001
Summer	8186 (24.31%)	128,145 (23.80%)	136,331 (23.82%)	
Autumn	8458 (25.12%)	146,884 (27.28%)	155,342 (27.15%)	
Winter	9032 (26.83%)	136,062 (25.27%)	145,094 (25.36%)	

**Table 2:** The descriptive summary of air pollutants and meteorological factors in the study area.

Pollutants (μg/m^3^)	Mean	SD	Min	Max	Percentiles		
					25th	50th	75th
**Trimester1**							
PM_2.5_	42.86	14.75	16.03	112.32	32.47	41.29	50.56
PM_10_	67.88	17.74	28.79	149.27	56.26	65.93	76.88
NO_2_	39.03	7.96	7.76	75.41	34.60	38.28	42.93
CO (mg/m^3^)	1.01	0.22	0.48	1.68	0.82	0.98	1.13
SO_2_	10.02	3.94	3.15	27.45	6.96	9.18	12.26
O_3_	37.78	19.69	3.70	110.68	19.74	36.41	52.51
Temperature (°C)	20.07	5.38	8.98	30.52	15.64	20.11	24.16
Relative humidity (%)	75.30	4.12	64.52	84.45	73.06	75.24	78.25
**Trimester2**							
PM_2.5_	41.76	14.16	16.59	107.25	31.08	40.08	49.93
PM_10_	66.49	17.27	28.41	147.33	54.94	64.22	76.54
NO_2_	38.86	7.47	9.75	73.61	34.81	37.95	42.89
CO (mg/m^3^)	1.02	0.23	0.48	1.68	0.83	1.00	1.14
SO_2_	9.61	3.60	3.21	25.46	6.84	8.76	11.68
O_3_	39.98	19.12	5.43	112.74	20.58	39.33	53.22
Temperature (°C)	20.31	5.04	9.85	29.09	16.13	20.32	24.37
Relative humidity (%)	75.27	3.81	66.78	82.87	72.81	75.24	78.00
**Trimester3**							
PM_2.5_	41.42	14.58	2.83	130.62	28.72	40.59	51.90
PM_10_	66.18	18.07	5.90	177.25	52.68	64.66	78.41
NO_2_	38.96	7.63	4.95	76.31	34.49	38.16	43.22
CO (mg/m^3^)	1.04	0.23	0.14	1.82	0.88	1.04	1.17
SO_2_	9.68	3.48	0.77	30.14	7.06	8.99	11.48
O_3_	37.34	19.16	2.38	34.61	18.84	37.16	51.28
Temperature (°C)	19.86	5.00	5.61	34.61	16.43	20.04	23.02
Relative humidity (%)	75.50	4.03	48.38	88.13	73.34	75.82	78.27
**Entire pregnancy**							
PM_2.5_	41.62	10.01	17.82	83.65	34.39	42.69	47.50
PM_10_	66.39	12.61	28.79	121.46	59.12	67.30	73.66
NO_2_	38.95	6.46	10.78	68.19	35.50	38.35	41.15
CO (mg/m^3^)	1.02	0.18	0.54	1.52	0.89	1.01	1.14
SO_2_	9.73	3.14	3.21	22.11	7.32	9.12	11.27
O_3_	38.45	13.34	8.27	105.65	30.24	39.07	47.38
Temperature (°C)	20.25	2.73	13.13	29.09	18.43	20.42	21.86
Relative humidity (%)	75.25	2.17	66.89	80.37	73.85	75.21	76.81

**Table 3:** Spearman correlation coefficients between ambient air pollutants and weather conditions.

Pollutants (μg/m^3^)	PM_2.5_	PM_10_	CO	NO_2_	O_3_	SO_2_	Temperature	Relative Humidity
PM_2.5_	1.000							
PM_10_	0.910 ***	1.000						
CO (mg/m^3^)	0.546 ***	0.527 ***	1.000					
NO_2_	0.476 ***	0.540 ***	0.400 ***	1.000				
O_3_	−3.390 ***	−0.272 ***	−0.449 ***	−0.281 ***	1.000			
SO_2_	0.519 ***	0.558 ***	0.381 ***	0.305 ***	−0.196 ***	1.000		
Temperature (°C)	−0.244 ***	−0.170 ***	−0.207 ***	−0.133 ***	0.378 ***	−0.121 ***	1.000	
Relative humidity (%)	−0.027 ***	−0.010 ***	0.104 ***	−0.047 ***	−0.326 ***	−0.136 ***	−0.441 ***	1.000

Note: Spearman correlation coefficients among daily average concentrations of air pollutants and meteorological factors in the study in Chongqing, China, 2015–2020. *** denotes *p* < 0.001.

**Table 4:** Adjusted relative risks (RRs) and corresponding 95% confidence intervals (CIs) from GAM models for PTB to maternal exposure to air pollutants by trimester of pregnancy.

Pollutant	Model	Trimester1		Trimester2		Trimester3		Entire Pregnancy	
		RR	95% CI	RR	95% CI	RR	95% CI	RR	95% CI
PM_2.5_	Model 1	0.958	(0.943, 0.973)	1.002	(0.984, 1.020)	**1.119**	**(1.101, 1.138)**	**1.185**	**(1.157, 1.215)**
	Model 2	0.988	(0.972, 1.004)	0.998	(0.980, 1.017)	**1.036**	**(1.021, 1.051)**	**1.101**	**(1.075, 1.128)**
	Model 3	0.987	(0.968, 1.006)	1.001	(0.979, 1.024)	1.008	(0.991, 1.024)	**1.064**	**(1.031, 1.099)**
PM_10_	Model 1	0.976	(0.963, 0.988)	0.940	(0.980, 1.008)	**1.107**	**(1.094, 1.121)**	**1.078**	**(1.059, 1.098)**
	Model 2	0.999	(0.986, 1.012)	0.976	(0.962, 0.989)	**1.027**	**(1.016, 1.038)**	**1.038**	**(1.020, 1.057)**
	Model 3	1.002	(0.985, 1.018)	0.998	(0.996, 0.999)	1.005	(0.992, 1.018)	1.004	(0.977, 1.030)
SO_2_	Model 1	0.733	(0.697, 0.771)	0.806	(0.764, 0.849)	0.897	(0.852, 0.945)	0.893	(0.840, 0.950)
	Model 2	0.960	(0.912, 1.010)	0.957	(0.908, 1.008)	1.014	(0.964, 1.067)	1.021	(0.960, 1.085)
	Model 3	0.988	(0.933, 1.047)	0.973	(0.915, 1.034)	1.011	(0.957, 1.068)	0.973	(0.901, 1.051)
NO_2_	Model 1	0.935	(0.920, 0.951)	0.919	(0.903, 0.936)	1.014	(0.997, 1.031)	0.886	(0.870, 0.903)
	Model 2	0.998	(0.981, 1.016)	0.999	(0.981, 1.017)	1.016	(0.999, 1.033)	0.982	(0.963, 1.002)
	Model 3	1.004	(0.986, 1.023)	1.002	(0.982, 1.022)	0.976	(0.959, 0.993)	0.991	(0.970, 1.013)
O_3_	Model 1	0.926	(0.914, 0.937)	0.922	(0.910, 0.934)	0.948	(0.937, 0.960)	0.921	(0.907, 0.934)
	Model 2	1.000	(0.988, 1.013)	0.996	(0.934, 1.009)	0.976	(0.966, 0.987)	0.987	(0.974, 1.001)
	Model 3	1.004	(0.990, 1.018)	1.004	(0.990, 1.019)	0.999	(0.988, 1.010)	**1.021**	**(1.002, 1.039)**
CO	Model 1	**1.857**	**(1.733, 1.991)**	**2.194**	**(2.035, 2.366)**	**1.758**	**(1.635, 1.890)**	**2.207**	**(2.031, 2.398)**
	Model 2	**1.081**	**(1.007, 1.162)**	**1.116**	**(1.035, 1.204)**	**1.167**	**(1.090, 1.250)**	**1.098**	**(1.011, 1.192)**
	Model 3	**1.094**	**(1.008, 1.188)**	**1.117**	**(1.024, 1.218)**	1.050	(0.978, 1.127)	0.878	(0.790, 0.976)

Note: Bold face indicates statistical significance established at *p* < 0.05 in the above three models. Model 1: single-pollutant model, adjusted for covariates including mean temperature and humidity; Model 2: single-pollutant model, adjusted for covariates including mean temperature and humidity, age of mother and father, and weight of birth; Model 3: multi-pollutant model, adjusted for covariates including mean temperature and humidity, age of mother and father, weight of birth, and additionally adjusted for other air pollutants.

## Data Availability

The datasets will be available on reasonable request from the first author.

## Supplementary Materials

The following supporting information can be downloaded at: https://www.mdpi.com/article/10.3390/ijerph19042211/s1, Table S1: Relative risks (RRs) and corresponding 95% confidence intervals (CIs) from GAM models for PTB to maternal exposure to air pollutants by trimester of pregnancy.
