# Supplementary material for: Association between Maternal Exposure to Ambient Air Pollution and the Risk of Preterm Birth: A Birth Cohort Study in Chongqing, China, 2015–2020
Source: Int J Environ Res Public Health. 2022 Feb 15;19(4):2211. doi: 10.3390/ijerph19042211 (PMC8871940; doi:10.3390/ijerph19042211)
Supplement: Supplementary file 1 [file ijerph-19-02211-s001.zip › ijerph-1520848-supplementary.pdf]

Supplementary Table S1. Relative risks (RRs) and corresponding 95% confidence intervals (CIs) from GAM models for PTB to maternal exposure to air pollutants by trimester of pregnancy.

Pollutant	Trimester1		Trimester2		Trimester3		Entire	
	RR	95%CI	RR	95%CI	RR	95%CI	RR	95%CI
$PM_{2.5}$	0.980	(0.966,0.993)	1.029	(1.014,1.043)	1.035	(1.020,1.050)	1.172	(1.150,1.195)
PM_{10}	0.988	(0.976,0.999)	1.017	(1.005,1.029)	1.036	(1.024,1.048)	1.117	(1.100,1.133)
SO_2	0.753	(0.716,0.791)	0.886	(0.842,0.931)	0.947	(0.899,0.998)	0.873	(0.822,0.928)
NO_2	0.942	(0.927,0.957)	0.936	(0.920,0.953)	0.949	(0.933,0.965)	0.933	(0.917,0.951)
O_3	0.931	(0.920,0.941)	0.930	(0.920,0.940)	0.951	(0.940,0.962)	0.894	(0.883,0.904)
CO	1.908	(1.781,2.043)	2.254	(2.094,2.427)	1.715	(1.597,1.842)	2.638	(2.429,2.864)
